# Biotin selective polymer nano-films

**DOI:** 10.1186/1477-3155-12-8

**Published:** 2014-03-21

**Authors:** Louise Elmlund, Subramanian Suriyanarayanan, Jesper G Wiklander, Teodor Aastrup, Ian A Nicholls

**Affiliations:** 1 Bioorganic & Biophysical Chemistry Laboratory, Centre for Biomaterials Chemistry, Linnæus University, Kalmar SE-391 82, Sweden; 2 Attana AB, Björnnäsvägen 21, Stockholm SE-114 19, Sweden; 3 Department of Chemistry - BMC, Uppsala University, Box 576, Uppsala SE-751 23, Sweden

**Keywords:** Molecularly imprinted polymer, Biotinylated compounds, Photoinitiated graft co-polymerization, Quartz crystal microbalance (QCM) sensors, Sandwich-casting method

## Abstract

**Background:**

The interaction between biotin and avidin is utilized in a wide range of assay and diagnostic systems. A robust material capable of binding biotin should offer scope in the development of reusable assay materials and biosensor recognition elements.

**Results:**

Biotin-selective thin (3–5 nm) films have been fabricated on hexadecanethiol self assembled monolayer (SAM) coated Au/quartz resonators. The films were prepared based upon a molecular imprinting strategy where *N,N'*-methylenebisacrylamide and 2-acrylamido-2-methylpropanesulfonic acid were copolymerized and grafted to the SAM-coated surface in the presence of biotin methyl ester using photoinitiation with physisorbed benzophenone. The biotinyl moiety selectivity of the resonators efficiently differentiated biotinylated peptidic or carbohydrate structures from their native counterparts.

**Conclusions:**

Molecularly imprinted ultra thin films can be used for the selective recognition of biotinylated structures in a quartz crystal microbalance sensing platform. These films are stable for periods of at least a month. This strategy should prove of interest for use in other sensing and assay systems.

## Background

Biotin is a water-soluble vitamin and enzyme cofactor recognized for its pivotal role in numerous metabolic pathways [1]. In humans and other mammals, it serves as a transient carrier of the carboxylate group and is involved in gluconeogenesis as well as fatty acid biosynthesis [2]. It is equally significant on account of its stable and strong interaction (*K*_d_ of ~10^-15^ mol/L) with the proteins avidin and streptavidin. This interaction has been exploited as an integral component in many biochemical assays and diagnostics [3,4], examples include methods based upon spectrophotometry [5], HPLC [6], radioligand binding [7], electroanalysis [8] and bioassays, *e.g.* ELISA [9]. Regeneration of assay materials requires disrupting the biotin-strept/avidin interaction, necessitating harsh treatment, *e.g*. elevated temperature (>70°C) [10]. Synthetic polymers capable of binding biotin could provide an interesting alternative as they are reusable and often able to withstand harsh conditions such as organic solvents and extremes of temperature, pH or ionic strength [11]. Biotin-selective materials that are amenable to autoclave treatment are of interest for use in conjunction with pull-down assays, *e.g*. from cell cultures. Several efforts have been made to design and synthesize discrete receptors with selectivity for biotin [12-16], and also biotin molecularly imprinted polymers [17-23].

Molecular imprinting technology [24-28] entails the preparation of polymer scaffolds for the selective binding of target molecules. The method makes use of stable complexes formed between functional monomers and template molecules. The complexes are fixed (imprinted) in a polymer matrix by initiating the polymerization reaction in the presence of a suitable crosslinking monomer. Subsequent removal of the template reveals cavities with structure and functionality complementary to the template.

Recent years have seen the development of strategies for preparing molecularly imprinted polymer (MIP) surfaces [29-31]. The combination of molecularly imprinted polymer films with piezoelectric transducers has been demonstrated useful for the development of chemosensors with sensitivities and selectivities necessary for application development [32-35].

**Figure 1:**
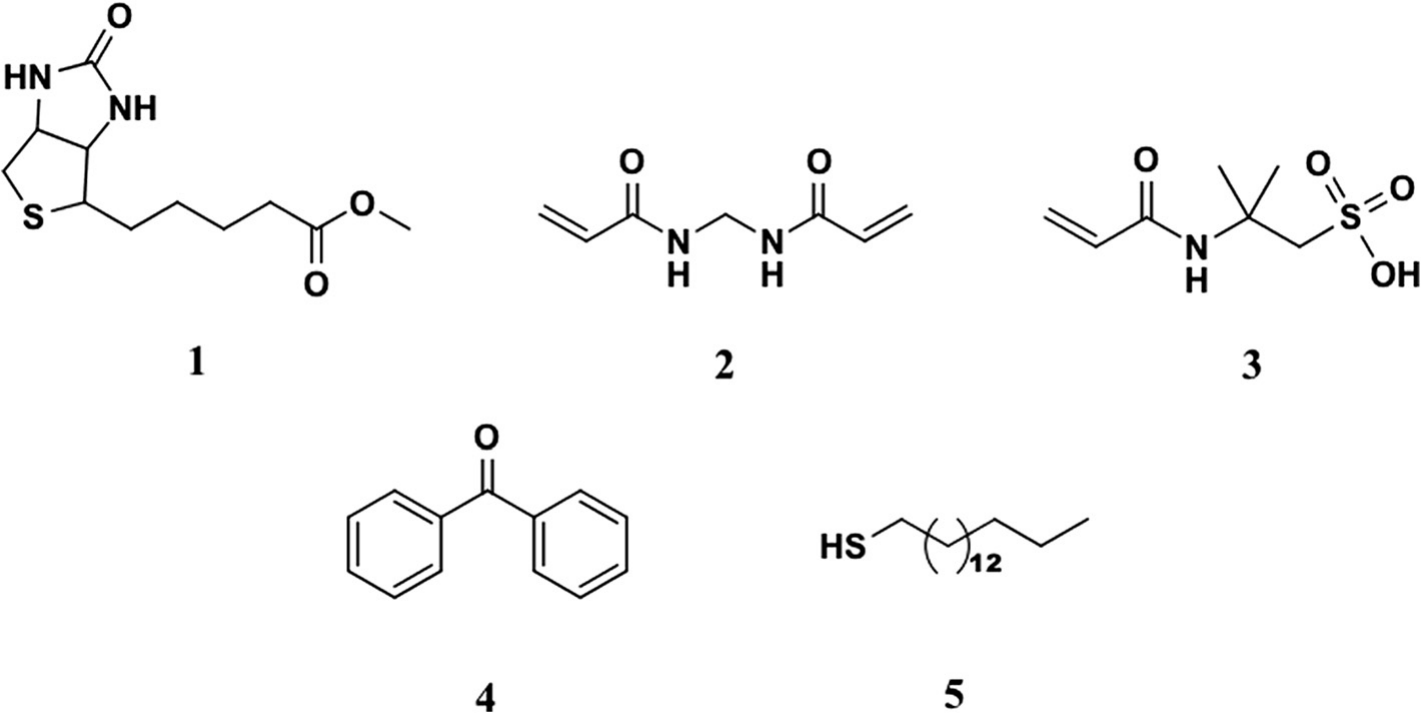
**Structures of chemicals used.** Biotin methyl ester (**1**), *N,N'*-methylenebisacrylamide (MBA, **2**), 2-acrylamido-2-methylpropanesulfonic acid (AMPS, **3**), benzophenone (**4**) and hexadecanethiol (HDT, **5**).

While good control of surface initiated polymer syntheses has been achieved using INIFERTER-based protocols [36], the additional synthesis steps and limits posed by substrate make more flexible initiation strategies desirable. Here we use the facile physisorption of benzophenone (**4**) on a self-assembled monolayer (SAM) of hexadecanethiol (HDT, **5**) as an initiator system for surface grafted polymer synthesis. Nanometer thick MIP films were prepared by the graft co-polymerization of *N,N*'-methylenebisacrylamide (MBA, **2**) and 2-acrylamido-2-methylpropanesulfonic acid (AMPS, **3**) in the presence of biotin methyl ester (BtOMe, **1**) (Figure 1). The selection of monomers was based upon the need for aqueous solubility and capacity to form relatively strong interactions with the template in aqueous media. As AMPS is deprotonated under the conditions present in the prepolymerization mixture, it was anticipated to afford ion-dipole interactions with the template. Even the water soluble crosslinker, MBA, is capable of acting as both a hydrogen bond donor and acceptor. The MIP and non-imprinted reference (REF) films were evaluated for thickness, topographical features and structural composition using a combination of ellipsometry, atomic force microscopy (AFM) and reflection absorption infrared spectroscopy (RAIRS). Quartz crystal microbalance (QCM) detection was employed to examine the recognition capabilities of the polymer films. The sensor characteristics, such as sensitivity, selectivity and stability constants for binding of biotin derivatives were determined under flow injection analysis (FIA) conditions.

## Results and discussion

**Figure 2:**
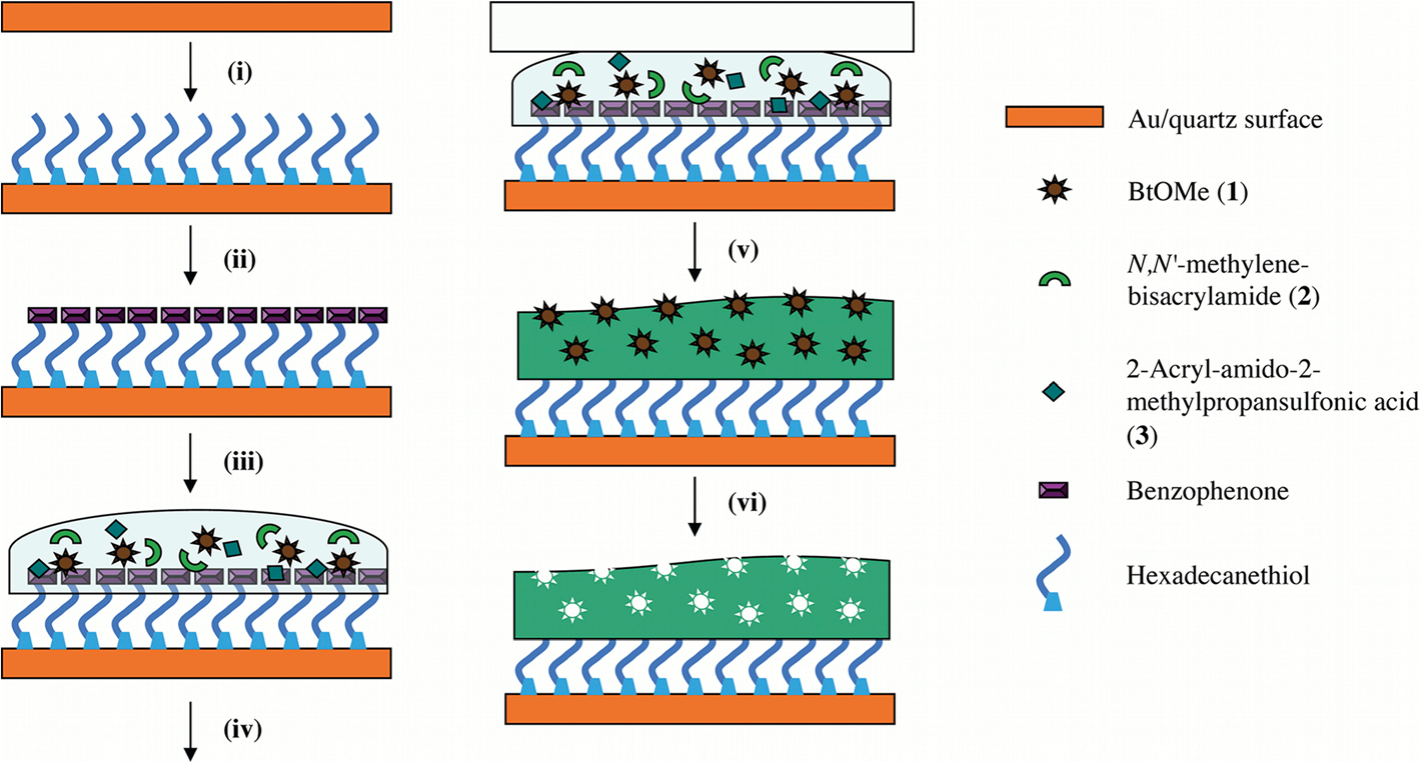
**Schematic illustration of molecularly imprinted polymer grafted SAM-Au/quartz nano-films. (i)** Self-assembly of hexadecanethiol on Au, **(ii)** physisorption of benzophenone photoinitiator, **(iii)** cover surface with pre-polymerization mixture, **(iv)** cover with glass slide, **(v)** irradiate with UV light and **(vi)** template extraction.

After optimization of the solvent composition for polymerization, biotin methyl ester imprinted and reference polymer films were grafted onto QCM sensor chips as shown in Figure 2. First, the gold surface was prepared for photografting polymerization by self-assembly of a monolayer of HDT followed by adsorption of the hydrophobic photoinitiator benzophenone. Next, the polymerization mixture containing template (BtOMe), functional monomer (AMPS) and cross-linker (MBA) in a 3:1 water–methanol mixture was added and subsequently subjected to UV-irradiation. The resultant polymer films were thoroughly washed before further examination.

**Figure 3:**
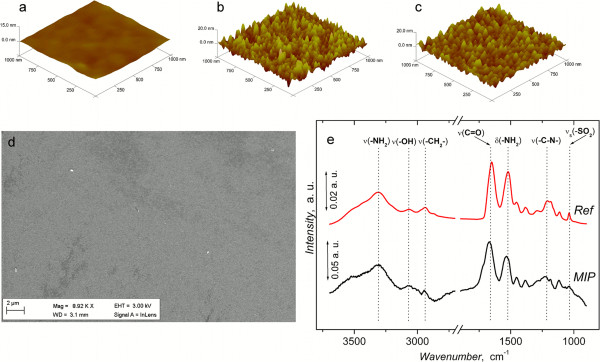
**Surface characterization of the polymer films.** Topography of **(a)** Au/quartz resonator **(b)** MIP and **(c)** REF polymer films measured using AFM. **(d)** Scanning electron micrograph of the REF polymer film. **(e)** RAIR spectra of MIP and REF polymer films.

AFM studies of the topographical features of the surfaces, Figure 3a-c, revealed that the SAM-Au/quartz surfaces were uniformly coated with MIP and REF films. The roughness values for the REF and MIP films were 20.6 and 20.3 Å, respectively. Ellipsometry was used to determine the average thicknesses of the films 33.95 ± 4.40 Å and 40.28 ± 12.40 Å (standard error of the mean) for MIP and reference polymers, respectively (n = 3). Importantly, as shown by SEM-studies, the polymer films were uniformly coated over the substrate (Figure 3d). FT-IR studies on the polymer films revealed that the MIP and REF copolymers both possessed functionalities corresponding to the monomers, MBA and AMPS (Figure 3e). Typical ν(N-H) stretching was observed as a broad peak at around 3300 cm^-1^. In addition, the bands arising from ν(C = O) and δ(N-H) of MBA were located at 1668 and 1531 cm^-1^, respectively. The presence of the sulfonic acid moiety (AMPS) can be inferred from the asymmetric as well as symmetric stretches of ν(SO_2_) and ν(O-H) at 1227, 1051 and 2954 cm^-1^.

**Figure 4:**
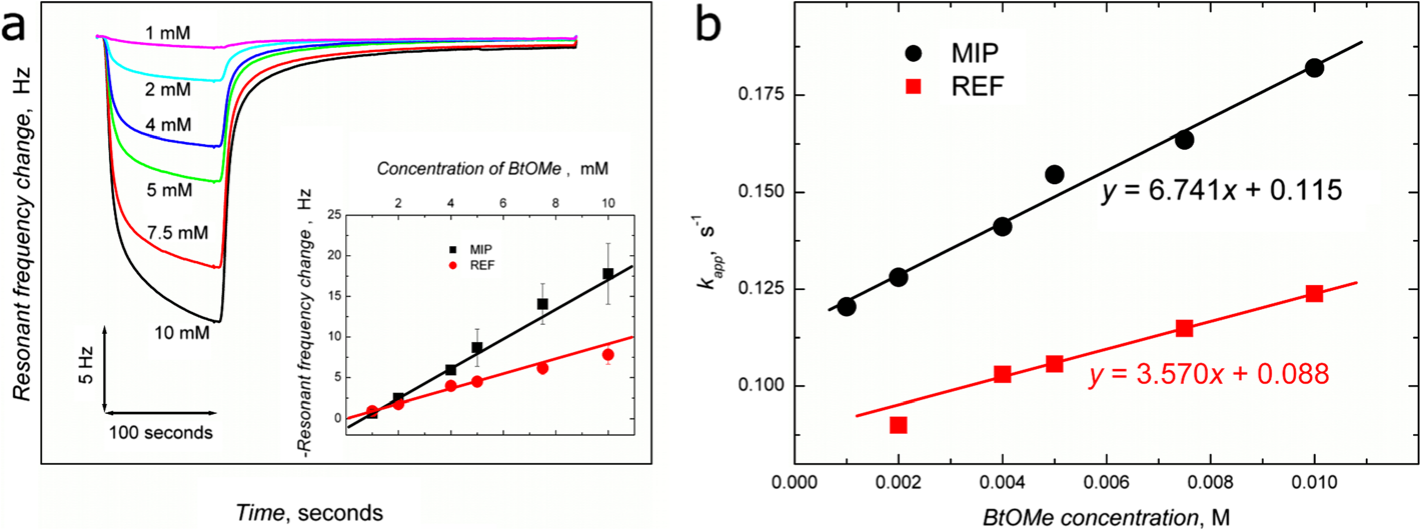
**Sensitivity and kinetics of polymer-ligand recognition. (a)** Resonant frequency change as a function of BtOMe concentration. Inset is the calibration plot for BtOMe on the MIP (*n* = 3, 6 injections per surface) and REF (*n* = 1, 6 injections) films. **(b)** Variation of observed *k*_app_ calculated from the associative part of the frequency vs time response curves for BtOMe binding to MIP and REF films .

The biotinyl moiety-selectivity of the MIP nano-films was examined by interrogating the films with a series of biotin derivatives while using the surfaces as QCM resonators under FIA conditions. Initial studies using **1**, Figure 4a, show a linear correlation between analyte concentration and maximum resonant frequency change. The response time (time taken by the signal to reach its 90% maximum value) for the sensor for template binding was as short as 32 s, and the sensor recovery time was ~ 4 min. As response and recovery times are influenced by ligand accessibility to the imprinted sites (mass transfer) and the strength of the MIP-analyte interaction, we conclude that the fast response and recovery times observed here are a direct result of the thinness of the films (3–5 nm) in conjunction with the presence of sites selective for the biotinyl moiety. The FIA calibration plot for the recognition of **1** by the polymer nano-films exhibits a linear relationship between sensor response (resonant frequency change, ΔF) and concentration of the injected analyte (Figure 4a, inset). The slope of the calibration curve is indicative of the sensitivity of the polymer for binding the template and includes both specific and non-specific binding. The sensor response for BtOMe was two times higher for the MIP-film than for the REF-film (Table 1).

**Table 1:** Sensitivity and stability constants of BtOMe templated-MIP and REF films, determined from QCM measurements

**Sensor/analyte**	**Sensitivity (Hz/mM) (*R***^***2***^**)**	**Stability constant, *K***_**s **_**(M**^**-1**^**)**
BtOMe on MIP film	1.8236 (0.9971)	58.54 ± 4.51
BtOMe on REF film	0.9157 (0.9959)	39.34 ± 2.12

For determination of the specific binding to the polymers, calculations used the initial part of the frequency response curves [37-39]. Analyte (A_n_) interaction with imprinted sites forms an affinity complex with the MIP film (MIP-A_n_). The mass of the MIP film increases upon analyte adsorption and the measured resonant frequency decreases sharply. The apparent rate constant, *k*_app_, for formation of the MIP-A_n_ complex was determined for each ligand concentration according to Skládal [38], Figure 4b. The slope and intercept of this plot gives *k*_a_ and *k*_d_, respectively. Calculation of the apparent stability constant (*K*_s_ = *k*_a_/*k*_d_) for BtOMe binding demonstrated a two-fold higher stability for binding to MIP than to REF (Table 1).

**Figure 5:**
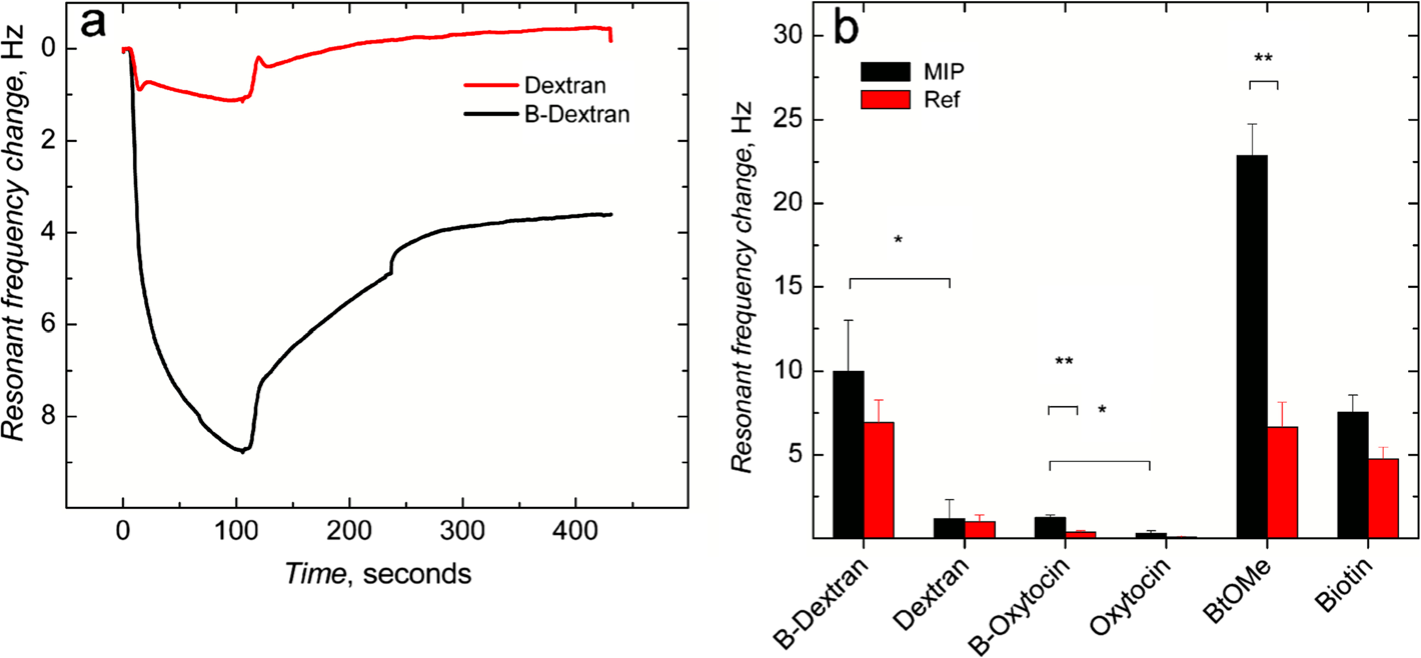
**Selectivity of polymer-ligand recognition. (a)** Frequency response for dextran and biotinylated dextran (B-Dextran) binding to the MIP film. **(b)** Resonant frequency changes for the binding of different analytes to MIP and REF films. * *p* > 0.05, ** *p* < 0.01, *n* = 3-8, 3–6 injections per surface.

**Figure 6:**
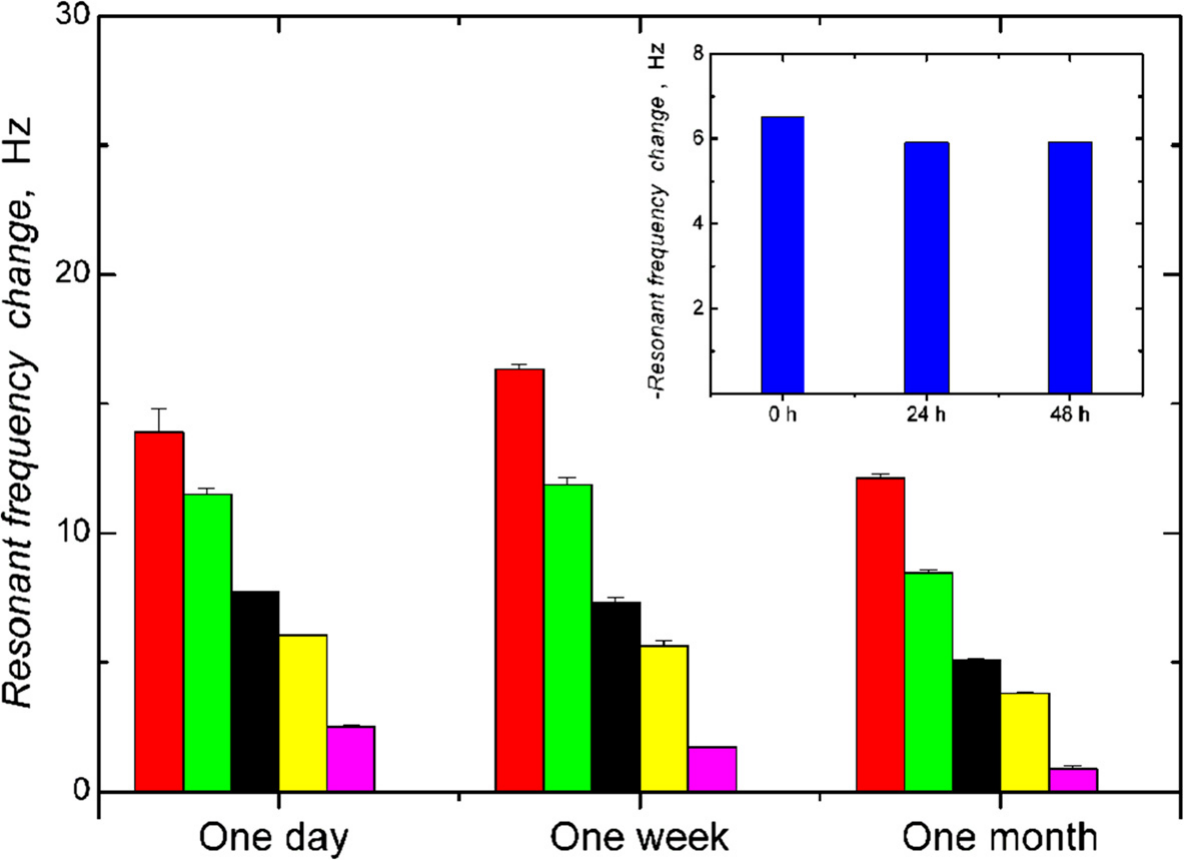
**Stability of polymer films.** Resonant frequency changes for the binding of BtOMe to the MIP film after prolonged storage in the dry state. Analyte concentrations used were 10 mM (red), 7.5 mM (green), 5 mM (black), 4 mM (yellow) and 2 mM (pink). Calibration was carried out on one MIP film at specified time intervals after fabrication (6 injections for one surface). Inset shows the sensor response for BtOMe (5 mM) injections under continuous flow (20 μL/min) after 0, 24, and 48 h.

To evaluate the selectivity of the MIP surfaces for the biotin-moiety, the surfaces were probed with a series of biotinylated substances (Figure 5). The QCM trace for 10 μM biotinylated- and non-biotinylated dextran (a carbohydrate) binding to the MIP surface, Figure 5a, illustrates the impact of the biotin moiety and the rapid response times. As seen in Figure 5b, the comparison of the behavior of the biotinylated and non-biotinylated derivatives upon interaction with the MIP- and REF-grafted SAM-Au/quartz resonator surfaces highlights the role of the biotinyl-moiety-selective sites. As also observed in the case of the peptide oxytocin, the biotinylated derivate showed higher selectivity than the non-biotinylated form. Moreover, the biotinylated forms demonstrate greater affinity for the MIP-surfaces than for REF-surfaces. Finally, the template (BtOMe) showed substantial preferential binding to the MIP, relative to the REF, the extent of which we suggest arises from this small structure being able to access more sites than in the case of the bulky carbohydrate and peptidic structures used here. The MIP film showed constant sensitivity toward BtOMe when stored under dry conditions at 4°C over a period of one month (Figure 6). In addition, no discernible difference in the frequency response was observed for BtOMe injections on the MIP film after continuous running in buffer (PBS) for 48 h (Figure 6, inset). Together these studies highlight the stability of the polymer films.

## Conclusions

Biotinyl moiety-selective molecularly imprinted polymer grafted SAM-Au/quartz nano-films of 3–5 nm thickness have been developed. The selectivity of these films for biotinylated structures has been demonstrated by employing the surfaces as piezoelectric resonators. Significant selectivity for biotinyl-bearing carbohydrate and peptidic structures was observed, and fast response and recovery times were achieved. The performance of these biotinyl-moiety-selective films was attributed to a combination of the Angstrom-level influence of the molecular imprinting process in conjunction with the nano-scale morphology of the polymer film. These results, together with the stability of these MIP-films, highlight the potential for application in sensor and functional material development.

## Methods

### Chemicals

All chemicals and solvents (HPLC grade) were obtained from commercial sources and used as received unless otherwise stated. BtOMe was synthesized as described in previous work [20]. MBA was purchased from Kodak (New Haven, CT). HDT, benzophenone, AMPS, biotin and dextran (analytical standard, Mw: 11,600) were from Fluka (Buchs, Switzerland). Biotin-dextran (Mw: 10000) was from Invitrogen (Eugene, OR). Biotin-oxytocin (Mw: 1235.5) was from AnaSpec, Inc (Fremont, CA). Oxytocin (Mw: 1000.19) and phosphate buffered saline (PBS) (tablets for preparing buffer containing 10 mM phosphate, 2.7 mM KCl and 137 mM NaCl, pH 7.4) were from Sigma (St Louis, MO). The water used was purified with a Milli-Q system (Millipore AB). Acetone and chloroform were dried over molecular sieves.

### Preparation of biotin methyl ester imprinted polymer films on Au/quartz resonator surfaces

#### SAM preparation and adsorption of initiator

AT-cut quartz resonators (QCM chips) of 7 mm diameter, 10 MHz fundamental frequency, sputtered with gold electrodes of 5 mm diameter and 140 nm thickness on each side (Attana AB, Stockholm, Sweden) were cleaned in fresh "piranha" solution (1:3 v/v mixture of 30% H_2_O_2_/conc. H_2_SO_4_) for 2 minutes, rinsed with plenty of water and washed three times in chloroform. *Caution: "Piranha" solution must be handled with extreme care since it is a hazardous oxidizing agent and reacts violently with most organic materials!* Self-assembled monolayers of HDT were prepared on the cleaned resonators by immersion in a 5 mM solution of HDT in dry chloroform over night. The resonators were then dried under a stream of N_2_, immersed in a 150 mM solution of benzophenone in dry acetone for 15 min and finally dried under a stream of dry N_2_.

#### Photoinitated graft co-polymerization

The polymerization method was adapted from earlier work using gold surfaces [40-42]. A solution of MBA (100 mM), AMPS (50 mM) and BtOMe (10 mM) in a water–methanol (3:1) mixture was sparged for 5 min with N_2_. 20 μl of the polymerization mixture was placed on top of the SAM-coated Au/quartz surface and covered with a piranha washed glass slide. This setup was placed under a UV lamp (254 nm, 35 mW/cm^2^) at 4°C for 15 min, without disturbing the assembly. After polymerization the resonators were subjected to a series of methanol (1 ml) washes (for at least 24 hours), until no evidence of template was observable by UV-spectroscopy. Finally, the polymer-coated Au/quartz resonators were dried under a stream of N_2_. Non-imprinted reference polymer films were prepared as described above though without the template.

### Characterization of biotin methyl ester imprinted polymer films on QCM crystals

#### AFM measurements

Topographical features of the polymer films were analyzed using AFM measurements performed on a Dimension 3100 SPM Instrument (Veeco industries, Plainview, NY) in the tapping mode using silicon probes. In this mode, the silicon probes were allowed to oscillate at their resonant frequency in order to avoid contact with the surface and improve the lateral resolution of the samples. The interaction force between the probe and sample, typically of the order 10^-9^ N, was maintained using a feedback loop. Any change in the force between the probe and sample affects the oscillation amplitude causing a vertical movement of the probe. An optical beam deflection setup monitoring vertical displacement of the probe followed the topography of the sample to be determined. The spring constant and the resonant frequency of the silicon probes were 40 N/m and 250 kHz, respectively. The sample surfaces were scanned (1 × 1 μm) at ambient laboratory temperature at a rate of 1 Hz. The sample roughness was evaluated as R_a_ (nm), computed using the software given by the instrument manufacturer.

#### Ellipsometry

The thickness of the polymer films was measured using a Rudolph Research AutoEl ellipsometer (USA) equipped with a He - Ne laser source (λ = 632.8 nm). The laser beam was reflected off the sample at an angle of incidence of 70° and the refractive index was assumed to be less than 1.5. Polymer thickness was averaged out from two measurements on each sample and repeated for three different samples prepared under analogous conditions. The polymer film thickness was calculated based on the optical constant of the bare gold surface, measured prior to preparation of polymer film, using the software provided by the manufacturer.

#### FT-IR

The imprinted and reference polymer films were characterized with RAIRS measurements using a Bruker Hyperion 3000 IR microscope with a built-in Tensor 27 IR spectrometer and a computerized sample stage. The IR beam was surface reflected twice at the surface with a grazing angle objective at 52° and 83° to the surface normal. A mercury-cadmium-telluride (MCT) detector was utilized to collect 1000 interferograms at 4 cm^-1^ resolution. Prior to Fourier transformation, the interferograms were corrected using a three-term Blackmann–Harris apodization function. The sample chamber was purged with N_2_ to maintain an inert atmosphere throughout the measurement. An unmodified gold-coated resonator surface was used as reference to measure the background spectra.

#### QCM measurements

The ligand binding characteristics of the polymer films were studied using a QCM biosensor (Attana Cell A200 instrument, Attana AB, Stockholm, Sweden) under FIA conditions. The experimental procedure can be summarized as follows: a continuous flow of phosphate buffered saline (PBS, 10 mM, pH 7.4) was passed through the QCM flow module at 20 μL/min until stabilization of baseline. Frequency changes from analyte-polymer interactions were measured after injection of 35 μL of analyte followed by 300 s of dissociation. Finally, to regenerate the surfaces, 35 μL of basic PBS buffer (pH 10.5) was injected followed by 600 s dissociation with PBS buffer. Data was collected and evaluated using Attester Evaluation software (Attana AB). For the selectivity studies, biotin methyl ester (10 mM), biotin (5 mM) and the biotinylated and non-biotinylated forms of dextran (10 μM) and oxytocin (25 μM) were used. The stability of the MIP film after storage under dry conditions at 4°C was assessed after 1, 7 and 30 days with respect to BtOMe sensitivity at different concentrations. The stability of the MIP film over a period of prolonged use under a continuous flow of PBS was tested using BtOMe (5 mM) injections after 0, 24 and 48 hours.

## Abbreviations

AFM: Atomic force microscopy; AMPS: 2-acrylamido-2-methylpropanesulfonic acid; BtOMe: Biotin methyl ester; FIA: Flow injection analysis; HDT: Hexadecanethiol; MBA: *N,N*'-methylenebisacrylamide; MIP: Molecularly imprinted polymer; QCM: Quartz crystal microbalance; RAIRS: Reflection absorption infrared spectroscopy; SAM: Self-assembled monolayer.

## Competing interests

The authors declare that they have no competing interests.

## Authors’ contributions

LE and JGW performed the thin-film syntheses. LE performed all ellipsometry and QCM studies. SEM, RAIRS and AFM studies were performed by SS and LE. All authors contributed to the project design, result analysis and drafting of the manuscript. All authors read and approved the final manuscript.
